# Recurrent Solitary Fibrous Tumor in Intradural Extramedullary Space: Case Report and Review of the Literature

**DOI:** 10.1155/2021/4559749

**Published:** 2021-11-20

**Authors:** Neris Dincer, Melisa Bagci, Metin Figen, Adem Yilmaz, Ahmet Mesrur Halefoglu, Canan Tanik, Esengul Kocak Uzel

**Affiliations:** ^1^Department of Radiation Oncology, Şişli Etfal Teaching and Research Hospital, Istanbul, Turkey; ^2^Department of Neurosurgery, Şişli Etfal Teaching and Research Hospital, Istanbul, Turkey; ^3^Department of Radiology, Şişli Etfal Teaching and Research Hospital, Istanbul, Turkey; ^4^Department of Pathology, Şişli Etfal Teaching and Research Hospital, Istanbul, Turkey

## Abstract

Solitary fibrous tumor/hemangiopericytoma (SFT/HPC) is a rare neoplasm arising from spindle cells and most commonly arising from pleura. Spinal SFT/HPC is a rare entity; hence, it is not on the top of the differential diagnosis list when a clinician faces a spinal lesion. In the review of the literature, there exist less than 50 case reports of intradural extramedullary SFT/HPC. Here, we present a 54-year-old female patient who underwent subtotal surgical excision of an intradural extramedullary spinal mass pathologically reported to be SFT/HPC and had symptomatic recurrence in the 3^rd^ year of follow-up. Surgical intervention was unachievable and the patient was given 45 Gy to the surgical cavity followed by a 5.4 Gy boost to visible tumor with external radiotherapy. Patient reported significant relief of her symptoms. We aim to contribute to the formation of a treatment algorithm for this rare entity.

## 1. Introduction

Solitary fibrous tumors (SFT) (formerly known as hemangiopericytoma (HPC)) are rare mesenchymal neoplasms originating from spindle cells [1]. Although the majority arise from pleura, 60% of SFTs originate outside the pleura [2], and SFTs are deemed to arise anywhere in the body. CD34 is a sensitive marker though it is not specific, and lately specific markers are identified [3]. Although surgery with negative margins is the mainstay treatment, the role of chemotherapy and radiotherapy is under debate [4]. This report presents a case of intradural extramedullary (IDEM) SFT in the thoracic spine.

## 2. Case Report

**Figure 1:**
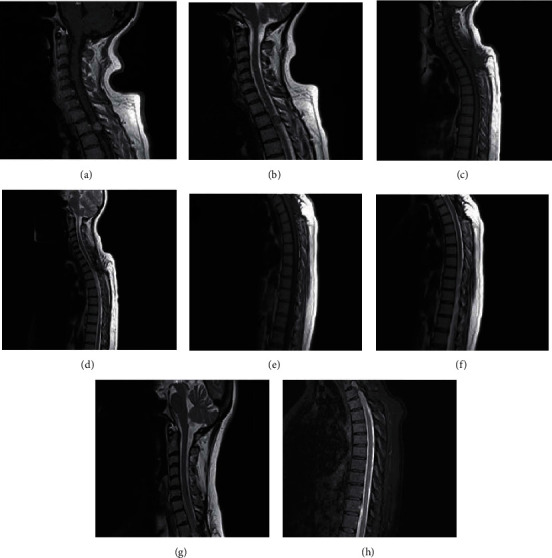
MRI images on preoperative, 3 months postoperative, 1 year postoperative, and current period. (a, b) (08.12.2017) Preoperative MRI reveals a lesion 12 × 10 mm in diameter, hypointense in both T1-w and T2-w, heterogeneously contrast enhancing with gadolinium. (c, d) (06.03.2018) Postoperative MRI reveals a paracentral mass located in the posterior edge of the spinal canal with minimal contrast enhancement. (e, f) (19.01.2019) Control MRI reveals. (g, h) (18.02.2021): MRI at current presentation reveals a lesion which is moderately hypointense in T1-w and hypointense in T2-w with homogeneous contrast enhancement.

**Figure 2:**
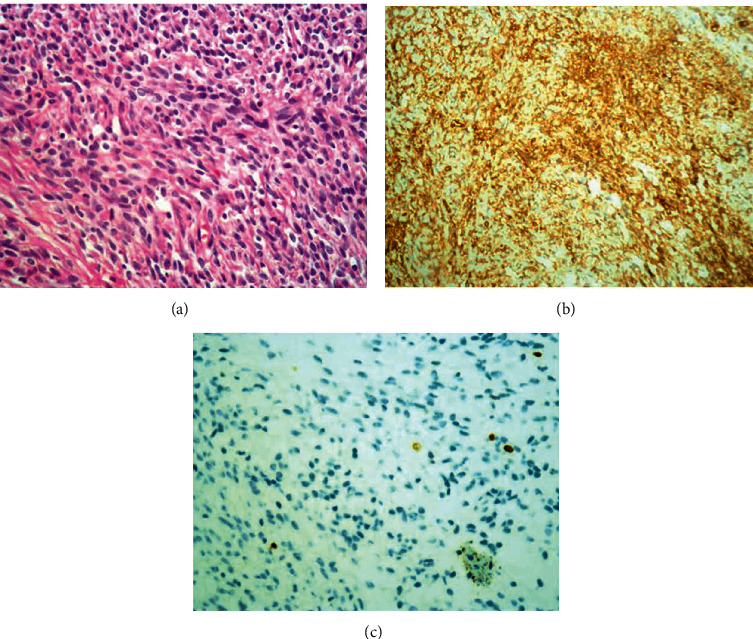
Pathology slides. (a) Hematoxylin and eosin staining of z200 magnification reveals spindloid nuclei and thin vascular structures. (b) Diffuse staining with CD34 at ×200 magnification. (c) 1-2% proliferation index with Ki-67 at ×200 magnification.

Our patient is a 54-year-old female with a past medical history including asthma and peptic ulcer disease. Her family history is free of malignant diseases. In 2016, patient's chest X-ray examination found a suspicious lesion. Magnetic resonance imaging (MRI) was ordered for further investigation in November 2017 since the patient was complaining of back pain. MRI revealed a spinal lesion 12 × 10 mm in diameter at the level of second thoracic vertebra (T2) (Figures 1(a) and 1(b)). The patient was referred to surgery and one month later underwent total laminectomy at the level of second thoracic vertebra (T2) and subtotal excision of intradural extramedullary lesion with neuromonitoring. The lesion involved nerve roots; complete resection could not be achieved due to perioperative neuromonitor signals. Postoperative neurological examination showed motor strengths as 3/5 for the left thigh, 3/5 for the left knee, 0/5 for the left foot, and 4/5 for the left hand. Patient was started on corticosteroid. Left lower extremity strength improved to 4/5, and the patient was discharged on postoperative day 4. A follow-up physical examination and MRI examination were planned for the patient 45 days after the surgery. Pathology report confirmed a WHO 2016 Grade 1 solitary fibrous tumor positive for CD34 and negative for S100, epithelial membrane antigen (EMA), and p53 (Figure 2). Thereafter, the patient underwent routine follow-up. MRI was performed 45 days after the surgery, and it was consistent with a remnant paracentral mass (Figures 1(c) and 1(d)). Control MRI a year after showed regression of the lesion (Figures 1(e) and 1(f)). In June 2020, no intraspinal mass lesion was revealed on MRI. In January 2021, the patient came for routine follow-up with a complaint of bilateral pain in her upper extremities as well as back pain. MRI examination of the patient was consistent with an intradural extramedullary lesion 10 × 5 mm in diameter which was compressing the spinal canal in the left paramedian area at the level of T2.

**Figure 3:**
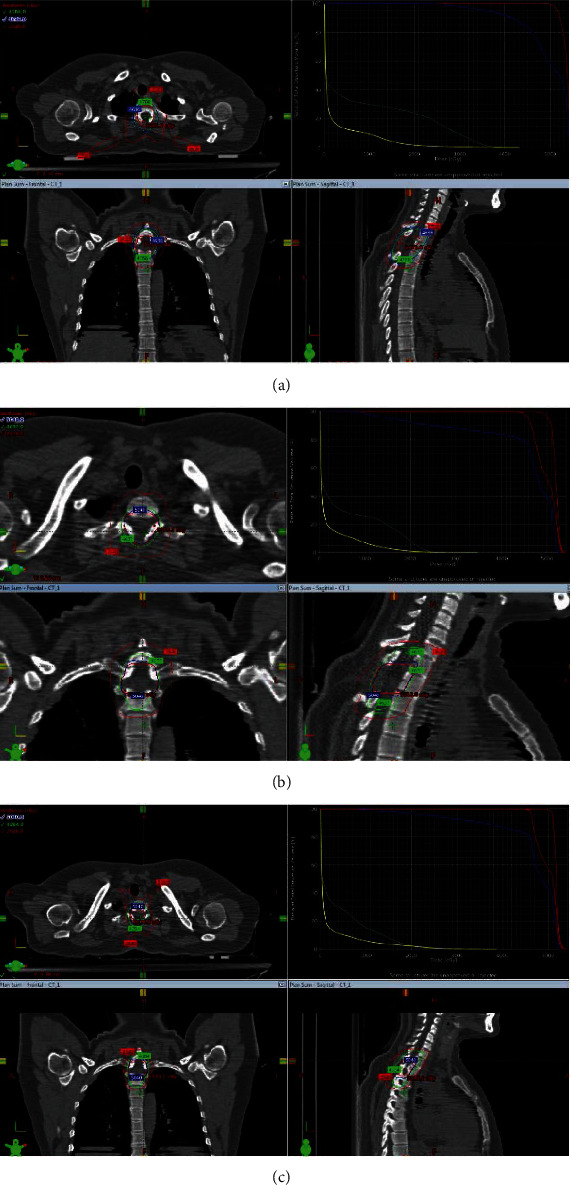
Plan summations and dose volume histogram (DVH) of 3DCRT, IMRT, and ARC. (a) Plan summations on axial (upper left), frontal (lower left), and sagittal (lower right) views and DVH graph (upper right) of conformal three-dimensional plan. Outer red line of plan summations represents 50% (25.2 Gy) isodose line, green line represents 42.84 Gy, and blue line represents 50.4 Gy. Red dots represent maximum point doses on each view. (b) Plan summations on axial (upper left), frontal (lower left), and sagittal (lower right) views and DVH graph (upper right) of intensity-modulated radiation therapy plan. Outer red line of plan summations represents 50% (25.2 Gy) isodose line, green line represents 42.84 Gy, and blue line represents 50.4 Gy. Red dots represent maximum point doses on each view. (c) Plan summations on axial (upper left), frontal (lower left), and sagittal (lower right) views and DVH graph (upper right) of volumetric modulated arc therapy plan. Outer red line of plan summations represents 50% (25.2 Gy) isodose line, green line represents 42.84 Gy, and blue line represents 50.4 Gy. Red dots represent maximum point doses on each view.

The patient was presented at the multidisciplinary tumor board for treatment options. Given the critical anatomic location and close involvement with nerve roots, the patient was found unsuitable for surgery. Eventually, the board decided on the referral to the radiation oncology clinic. A repeat contrasted thin-slice MRI of cervical and thoracic spine was performed (Figures 1(g) and 1(h)). The report confirmed the recent findings. Patient underwent 1 mm thin slice treatment-planning computer tomography (CT) with intravenous contrast infusion. CT-MRI image fusion was acquired with the preoperative and present MRI for better discrimination of the resection cavity and the boundaries of the present lesion to achieve improved target delineation for treatment planning [5]. Gross tumor volume (GTV) was defined as the postoperative surgical cavity and clinical target volume (CTV) was extended 2 cm above and below the GTV. Planned RT dose was 45 Gray (Gy) in 25 fractions (1.8 Gy/fraction) and a boost of 5.4 Gy in 3 fractions (1.8 Gy/fraction). Organs at risk (OAR) were determined as the spinal cord, lungs, and esophagus in accordance with the atlas of dose constraints in thoracic radiotherapy published by Kong et al. [6]. Maximum point dose constraint to the spinal cord was determined to be 45 Gray (Gy) in accordance with the Quantitative Analyses of Normal Tissue Effects in the Clinic (QUANTEC) model to avoid any radiation-related side effect, namely, myelopathy [7]. Treatment planning was done with conformal three-dimensional (3D), intensity-modified radiation therapy (IMRT), and volumetric modulated arc therapy (VMAT, hereinafter referred to as ARC) approach, respectively (Figure 3). QUANTEC normal tissue tolerances were taken into consideration and the calculations were made accordingly (Table 1) [8]. ARC was the chosen modality with optimal spinal cord protection compared to 3D and tolerable treatment duration for patient comfort and cooperation to stand immobile during the treatment compared to IMRT.

## 3. Discussion

Primary spinal cord tumors are rare neoplasms that constitute 4.5% of central nervous system (CNS) tumors in adults [9]. Their primary treatment is surgical resection if possible [10], and radiotherapy can ensue if total resection is not achieved [11]. They are classified according to their location as extradural, intradural extramedullary and intramedullary. Extradural tumors are most common followed by intradural extramedullary (IDEM) tumors. Meningioma, schwannoma, and neurofibroma comprise the majority of intradural extramedullary tumors [12]. Solitary fibrous tumors of intradural extramedullary space is a rare entity. They correspond to the group IIA in the classification of Liu et al. [13] (Table 2). The majority of type IIA spinal SFTs arise at the thoracic spine [12].  Table 3 summarizes the cases present in the PUBMED database.

Due to their rareness, SFTs are not on the top of the differential diagnoses list when the clinician is faced with a lesion in the spinal cord [14]. MRI is the preferred modality to delineate the tumor and evaluate for invasion, but the imaging findings are variable and nonspecific [15]. Nevertheless, the diagnosis is less of a challenge with the advances in knowledge in pathological markers. CD34, although not specific, is deemed to be the histological hallmark of SFTs and has been found to be expressed in 79% of cases [16, 17]. A retrospective study with 16 SFTs located in the spinal cord confirmed this finding with 100% SFTs being positive for CD34 and negative for EMA, GFAP, and MBP staining. S-100 positivity varied with 5 of the patients staining positive [18]. Lately, STAT6 and GRIA2 are proposed to be distinguishing markers for SFTs [19, 20]. STAT6 expression is driven by NAB2-STAT6 gene fusion, and STAT6 has lately been sought to be a specific marker for SFT [19, 21, 22]. STAT6 positivity in SFTs and HPCs is considered as finding that supports the abolishment of the thin boundary between these 2 entities in late 1990s [3, 23, 24].

Achieving complete surgical resection is the main goal in extrathoracic SFTs, and it is associated with improved local control and survival. Routine long-term follow-ups must be ensured for the early detection of recurrence [25]. Although there are no standardized follow-up routines for these patients. The anatomical location of the tumor may not allow the total resection in some cases [4]. The role of radiotherapy is under debate in SFTs. There are studies advocating that RT is not strictly indicated after complete resection due to close follow-up and low recurrence rate [4] and that adjuvant radiotherapy can be considered in the case of subtotal resection [26–28] or if we are facing a high grade SFT [29]. There are studies advocating adjuvant radiotherapy be standard of treatment [18, 30]. Complete resection, low-grade tumor and young age were reported to be factors that led the clinicians to omit radiotherapy [31]. Krengli et al. revised 151 extrathoracic SFT patients while Wang et al. reviewed 16 spinal SFT patients in terms of the effect of GTR vs. GTR+RT on local recurrence (LC), disease-free survival (DFS), and overall survival (OS), and they both came up with the same conclusion: addition of adjuvant RT improved LS and DFS while GTR was the main predictor of OS [18, 32]. While a retrospective review reveals that the median radiation dose for extracranial SFTs is 60 Gy [32], this dose seems to be unachievable in the spinal SFTs due to dose constraints of the spinal cord, which is 45-50 Gy. Retrospective single center study of Wang et al. reported a median dose of 40 Gy in patients with spinal SFT [18]. The treatment plan must be tailored according to location and extent of the tumor. Radiation doses may vary in between patients if the plan cannot limit spinal cord doses in the desired limits.

Herein, we present a case of IDEM SFT who had STR 27 months prior to presentation to our clinic with bilateral pain over the arms and legs and newly developed radiological evidence of recurrence. Radiotherapy was the treatment of choice for this patient due to the location of the tumor. To our knowledge, we present the 5^th^ case of a recurrent SFT treated with radiotherapy [18, 33–35].

## 4. Conclusion

Spinal type IIA SFT is a rare entity with less than 50 case reports in the literature. GTR is the required treatment option while the role of adjuvant radiotherapy and its indications is yet to be discussed. We contribute to the literature by presenting a rare case in which close follow-up ensued STR, and radiotherapy was performed when the lesion recurred. We believe that the increase in the number of cases in the literature will help and contribute to the embodiment of the therapeutic algorithm of the disease in question.

## Figures and Tables

**Table 1:** Comparison of treatment plans.

	PTV			Spinal cord	Lung			Esophagus		Monitor unit (MU)
	D50 (cGy)	D98 (cGy)	D2 (cGy)	D2 (cGy)	V20 (%)	V5 (%)	Mean (cGy)	33% (cGy)	Mean (cGy)	
3D	5370	5090	5468	5437	1.7	12.5	230	839	562	455
IMRT (5 field)	5182	5067	5302	5226	2	10	190	385	255	1353
ARC	5200	4950	5333	5220	1	12	207	540	326	1060

3D: conformal three-dimensional; IMRT: intensity-modified radiation therapy; ARC: volumetric modulated arc therapy; PTV: planning target volume; D95: dose covering 95% of the PTV; D50: median dose, D98: near-minimum dose, D2: near-maximum dose; cGy: centigray; V20: lung volume that received a dose of 20 Gy or more; V5: lung volume that received a dose of 5 Gy or more; 33%: dose received by 33% of esophagus.

**Table 2:** Liu classification for localization of spinal SFTs [13].

Type	Subtype
Type 1: extradural	IA, intracanal type
	IB, intra- and extracanal type
Type II: intradural	IIA, extramedullary type
	IIB, intramedullary invasion type
Type III: intra- to extradural and paravertebral type	

**Table 3:** Reported cases of spinal intradural extramedullary solitary fibrous tumor/hemangiopericytoma.

Case report, year	Age/sex	Chief complaint at presentation	Level	T1-W	T2-W	Contrast enhancement	Treatment	RT regimen (if performed)	Outcome	Treatment of recurrence
Pitlyk et al., 1965 [36]	39/M	Total paraplegia	T8	N/A	N/A	N/A	GTR	N/A	Tumor recurred 4 times in 8 years	Reoperation
Pitlyk et al., 1965 [36]	60/M	Paresthesia and weakness of RL	C4	N/A	N/A	N/A	GTR	N/A	N/A	
Pitlyk et al., 1965 [36]	49/F	Left-sided weakness and paresthesias	C3	N/A	N/A	N/A	GTR	N/A	10 years NED	—
Kitanaka et al., 1993 [37]	59/F	Gait disturbance	T6	Isointense	N/A	HomE	EBR	N/A	16 months NED	
Malek et al., 1997 [38]	33/M	Back pain Progressive myelopathy LE dysesthesias	T7-T8	—	Hypointense	Mild enhancement	Resection, NS	N/A	N/A	
Kanahara et al., 1998 [39]	62/M	Sensory disturbance of bilateral lower extremity	C6-C7	Hypointense	Hypointense	Marginal enhancement	GTR	N/A	N/A	
Brunori et al., 1999 [40]	46/F	Left sciatalgia	T12-L1	T1 görüntü	NS	HomE	GTR	N/A	4 months NED	
Vorster et al., 2000 [41]	51/M	Burning sensation and numbness in both thighs weakness in the LLE	T2-T3	Well-circumscribed mass at T2–3, which enhanced homogeneously on T1-weighted MR imaging with gadolinium	NS	HomE	GTR	N/A	7 months NED	
Kurtkaya et al. 2001 [42]	70/F	RL weakness	T3	Irregular isointensity	Irregular hypointensity	E	CR	N/A	No recurrence until the report	
Dufour et al., 2001 [43]	45/M	Paraparesis	Cervical (not specified)	N/A	N/A	N/A	CR	N/A	2 years NED	
Dufour et al., 2001 [43]	18/F	Dorsalgia	Thoracal (not specified)	NM	N/A	N/A	STR + RT	40 Gy	Alive after 4.6 years of f/u	
Dufour et al., 2001 [43]	43/F	Intercostal neuralgia	Thoracal (not specified)	NM	N/A	N/A	CR	N/A	4.1 years NED	
Dufour et al., 2001 [43]	38/M	Intercostal neuralgia	Thoracic (NS)	N/A	N/A	N/A	CR + RT	40 Gy	12.8 years NED	
Betchen et al., 2002 [44]	31/M	B/L leg paresthesia LE cramping and fatigue	L4	Intermediate signal	Increased signal	Diffuse enhancement	EBR	N/A	No recurrence at the time	
Pizzolitto et al., 2004 [45]	36/M	Paresthesias of right foot LE weakness Urinary loss and rectal incontinence	T7/T8	NS	NS	HomE	CR	N/A	12 months NED	
Bohinski et al., 2004 [46]	49/F	Neck pain and stiffness	C4	İsointense	Hypointense	HomE	EBR	NA	10 months NED	
Piana et al., 2004 [47]	67/M	Lumbar pain Weakness in RLE	L1-L2	NS	NS	HetE	GTR	—	—	—
Ogawa et al., 2005 [48]	63/F	Sensory disturbance of LE	T11	Mildly hypointense	Hypointense	HomE	GTR	—	18 months NED	—
Pakasa et al., 2005 [49]	27/M	Back pain	T5-T7	—	—	—	STR	—	Recurrence after 14 years	Surgery
Munoz et al., 2008 [33]	35/M	Hypoesthesia	Sacral	—	—	—	CR	—	Pulmonary mets at 4th year	Observation
									New nodules and lumbosacral recurrence at the 6th year	Palliative RT
									New metastatic lesions in lungs and liver at the 7th year	Chemotherapy
Fitzpatrick et al., 2009 [50]	54/M	Left paraspinal, buttock, and anterolateral thigh pain radiating down the anterior shin and left foot	L4-L5	Soft tissue signal intensity	Soft tissue signal intensity	NS	GTR + RT	NS	N/A	
Arantes et al., 2009 [22]	22/M	Dorsal pain associated with B/L hypoesthesia and weakness of the LE	T1-T2	Isointense	Isointense	HomE	GTR	—	18 months NED	
Moscovici et al., 2011 [51]	20/M	Mild thoracic back pain radiating to the LL progressive paraparesis fecal and urinary incontinence	T9-T10	Isointense	Isointense	HomE	GTR Patient was referred to RT but declined and went with imaging and clinical follow-up	Patient declined the treatment	2 years NED	
Ackerman et al. 2011 [52]	58/M	Acute weakness of the LE bladder and bowel incontinence	T10	Isodense	Hyperdense	E	EBR	—	N/A	
Kirkbride et al., 2011 [53]	72/M	Acute urinary retention	C3	NS	Moderately hyperintense	HomE	CR	—	—	—
Bisceglia et al. [54]	47/M	Weakness in RLE	T3-T4	NS	NS	—	GTR	—	11.6 years NED	—
Brigui et al., 2012 [55]	56/M	Neuropathic pain of the RLE LL pain and gait difficulties	T6-T7	Isointense	Isointense	HomE	GTR	—	29 months NED	
Mariniella et al., 2012 [56]	67/M	Pain dysesthesia and weakness in left arm and leg	C4-7	Isointense	Hypointense	HomE	GTR	—	1 year NED	
Shirzadi et al., 2013 [31]	49/M	Patient with a history of SFT presented with left LE weakness and B/L radicular pain	T9-T12	NS	NS	E	Debulking	—	Recurred in postoperative month 4 with multiple metastases	No intervention
Shirzadi et al., 2013 [31]	57/M	Lower back pain	T9-T10	NS	NS	E	Preoperative embolization followed by microdissection + adjuvant RT	(IMRT of 5580 cGy over 48 elapsed days) to his resection cavity	3 years NED	
Shirzadi et al., 2013 [31]	56/M	Neck discomfort radiating into head and both shoulders	C1-C3	NS	NS	E	GTR	Radiosurgery was performed on recurrence (month 6- T2-3 level)	3 years NED	
Drazin et al., 2013 [34]	56/M	Neck pain radiating to head and shoulders	C0-C4	NS	NS	E	CR	—	Metastasis at T2-T3 6 months postoperative	Radiosurgery
Lee et al., 2013 [57]	21/M	Neck pain B/L tingling sensation in both hands	C1-C2	Isointense	Hyperintense	HomE	CR	—	1 year NED	
Kobayashi et al., 2014 [58]	40/M	Gait disturbance and numbness in both hands	C4-C5	Hypointense	NS	HomE	Resection, NS	—	Recurrence after 17.7 years	STR
Sade et al., 2015 [59]	43/M	Weakness of LE		NS	Hypointense	HomE	Resection, NS	N/A	NM	
Das et al., 2015 [60]	50/M	Quadriparesis, numbness in B/L UE	C4-C5	NS	NS	E	GTR + adjuvant RT (EBRT) + adjuvant CT	NS	23 months NED	
Das et al., 2015 [60]	12/F	Paraparesis	T11-L1	NS	NS	E	GTR + RT	NS	9 months NED	
Kaur et al., 2015 [11]	16/M	Loss of strength in LE	T9	N/A	N/A	N/A	Excision, NS followed by RT	f 45 Gy in 25 fractions over a period of 5 weeks	5 years NED	
Basaran et al., 2015 [61]	67/M	Walking disability	L3	NS	Hypointense	HomE	Surgery, NS	—	12 months NED	
Biswas et al., 2017 [62]	35/F	Backache LE weakness	T10-T11	Hypointense	Hyperintense	NM	STR + metastasectomy	20 Gy in five fractions over 1 week to the primary residual site		
Chew et al., 2017 [63]	63/M	Back pain Paraparesis Sensory deficit of LE	T9	NS	NS	E	GTR	N/A	12 months NED	
Albert et al. 2017 [64]	10/M	RUE weakness	C1-C3	Hypointense	Hypointense	HomE	GTR	—	1 year NED	—
Tomamatsu et al., 2019 [65]	68/F	Incidental lesion on imaging	T9	Isointense	Hypointense	HetE	GTR	N/A	3 years NED	
Wang et al. 2019 [18]	21/M	Backache	T3-T4	NS	Slightly hyperintense	NS	GTR + RT	Not specified for the patient; median dose is 40 Gy (35-45 Gy)	96 months NED	
Wang et al. 2019 [18]	43/F	Backache	T8	NS	Slightly hyperintense	NS	GTR + RT	Not specified for the patient; median dose is 40 Gy (35-45 Gy)	Recurrence 49 month postoperatively	RT
Wang et al. 2019 [18]	35/F	Neck pain UE numbness	C4-C6	NS	Slightly hyperintense	NS	STR	N/A	Recurrence at month 32 postoperatively	No treatment
Wang et al. 2019 [18]	33/F	Backache	T9	NS	Slightly hyperintense	NS	GTR + RT	Not specified for the patient; median dose is 40 Gy (35-45 Gy)	70 months NED	
Wang et al. 2019 [18]	57/M	Backache	T3-T4	NS	Slightly hyperintense	NS	GTR	N/A	67 months NED	
Wang et al. 2019 [18]	37/F	Backache	T6	NS	Slightly hyperintense	NS	GTR	N/A	Recurrence at month 22 postoperatively	RT
Wang et al. 2019 [18]	40/F	Backache LE weakness	T11-t12	NS	Slightly hyperintense	NS	STR + RT	Not specified for the patient; median dose is 40 Gy (35-45 Gy)	42 months NED	
Wang et al. 2019 [18]	49/M	Backache	T4	NS	Slightly hyperintense	NS	GTR	N/A	35 months NED	
Wang et al. 2019 [18]	34/F	Neck discomfort	C6-c7	NS	Slightly hyperintense	NS	GTR	Not specified for the patient; median dose is 40 Gy (35-45 Gy)	24 months NED	
Murata et al., 2020 [35]	49/F	Chest pain	T6	İsointense	Hypointense	NS	GTR	—	Recurrence at 12 month postoperatively	Carbon ion radiotherapy-64 Gy
Kim et al., 2020 [66]	64/M	RL pain		Hypointense	Hypointense	HetE	Surgical removal, NS	N/A	1 year NED	
Glauser et al., 2020 [67]	72/M	LE weakness, pain and numbness	C5-C7	T1w hypointense (?)	NS	NS	GTR	N/A	N/A	
Singla et al., 2020 [68]	50/M	Quadriparesis, numbness in B/L UE	C4-C5	N/A	N/A	N/A	GTR + adjuvant RT + adjuvant CT	Not specified	Recurrence at 62 months postoperatively	
Singla et al., 2020 [68]	12/F	Acute conus cauda syndrome	T11-L1	N/A	N/A	N/A	GTR + adjuvant RT	NS	52 months NED	
Singla et al., 2020 [68]	38/M	Spastic paraplegia bladder dysfunction	C7-T1	N/A	N/A	N/A	GTR	N/A	50 months NED	
Koduru et al., 2020 [69]	16/F	Weakness of RUE and RLE	C5-C7	Hypointense	Hyperintense	HomE	CR	N/A	N/A	
Dauleac et al., 2020 [70]	67/M	Asthenia and weakness RLE	T8-T9	NS	Hyposignal	NS	CR	N/A	1 year NED	

Abbreviations are as follows: M: male; F: female; LE: lower extremity; RLE: right lower extremity; LLE: left lower extremity; UE: upper extremity; RUE: right upper extremity; LUE: left upper extremirt; LL: left leg; RL: right leg; B/L: bilateral; C: cervical; T: thoracal vertebra; L: lombar vertebra; HomE: homogenous enhancement; HetE: heterogenous enhancement; E: enhancement (not specified) GTR: gross total resection; STR: subtotal resection; CR: complete resection; EBR: en bloc resection; NS: not specified; RT: radiotherapy; CT: chemotherapy N/A: not applicable NED: no evidence of disease; Gy: gray.
